# Aerobic Glycolysis in the Retina: Functional Roles of Pyruvate Kinase Isoforms

**DOI:** 10.3389/fcell.2020.00266

**Published:** 2020-04-30

**Authors:** Raju V. S. Rajala

**Affiliations:** ^1^Department of Ophthalmology, The University of Oklahoma Health Sciences Center, Oklahoma City, OK, United States; ^2^Department of Physiology, The University of Oklahoma Health Sciences Center, Oklahoma City, OK, United States; ^3^Department of Cell Biology, The University of Oklahoma Health Sciences Center, Oklahoma City, OK, United States; ^4^Dean McGee Eye Institute, Oklahoma City, OK, United States

**Keywords:** pyruvate kinase M2, pyruvate kinase M1, posttranslational modifications, photoreceptor cells, glycolysis, Warburg effect, anabolic processes

## Abstract

One hundred years ago, Otto Heinrich Warburg observed that postmitotic retinal cells are the highest oxygen-consuming cells in the body. He compared these cells to actively growing mitotic tumor cells since both cells reprogram glucose for anabolic processes, which include lipid, protein, and RNA/DNA synthesis, and for antioxidant metabolism. To achieve this metabolic reprogramming, cancer cells preferentially express a less active dimeric form, the M2 isoform of pyruvate kinase (PKM2), which shuttles glucose toward the accumulation of glycolytic intermediates that redirect cell activities into anabolic processes. Similar to cancer cells, retinal photoreceptors predominantly express the M2 isoform of PKM2. This isoform performs both metabolic and non-metabolic functions in photoreceptor cells. This review focuses on the metabolic and non-metabolic roles of pyruvate kinases in photoreceptor cell functions.

## Introduction

Almost 100 years ago, German physiologist Otto Heinrich Warburg noticed that retina consumes the highest amount of oxygen in the body; he compared this oxygen consumption to that of a rapidly dividing cancer cell (Warburg, 1956a,b). The Warburg effect is described as a reprogramming of cancer cell’s metabolism such that the cell uses more glucose than does a normal cell and redirects the glucose for use in anabolic processes, such as lipid, RNA/DNA synthesis, and NADPH generation, which results in incomplete glucose oxidation in the presence of oxygen (Warburg, 1956a). This phenomenon is also called aerobic glycolysis. The difference between a cancer cell and retinal cells, especially the highly oxygen-consuming photoreceptor cell, is mitotic versus post-mitotic. The Warburg effect is observed in fetal cells, rapidly proliferating tumor cells, and retinal cells (Warburg, 1956a,b; Winkler, 1981; Fiske and Vander Heiden, 2012; Casson et al., 2013; Ng et al., 2014; Rajala and Gardner, 2016). The question becomes why a non-dividing photoreceptor cell needs a Warburg effect. Two types of photon-absorbing preceptors are present in the retina: rods and cones. Rods are mainly used in dim light, whereas cones are needed for daylight color vison (Soucy et al., 1998). The photoreceptor is surrounded by a plasma membrane in which disc membranes are loaded with a light-absorbing G-protein coupled receptor, rhodopsin. Every day, by the onset of light, 10% of outer segment tips are engulfed by the neighboring retinal pigment epithelial (RPE) cells (LaVail, 1976), and some of the digested products are recycled back to photoreceptor and other cells of the retina (Bok, 1985). The RPE engulfment of photoreceptors is called phagocytosis. Also, the RPE continuously provides essential nutrients, especially glucose through glucose transporters, and oxygen to photoreceptor cells for survival and maintenance (Kanow et al., 2017). Daily phagocytosis leaves a gap between the photoreceptors and the RPE. New membrane synthesis (disc biogenesis) has been very efficient to maintain the length of the photoreceptor cells for proper RPE-photoreceptor interaction (Punzo et al., 2012). It was suggested that aerobic glycolysis might be essential for photoreceptor membrane biosynthesis (Punzo et al., 2012).

**FIGURE 1:**
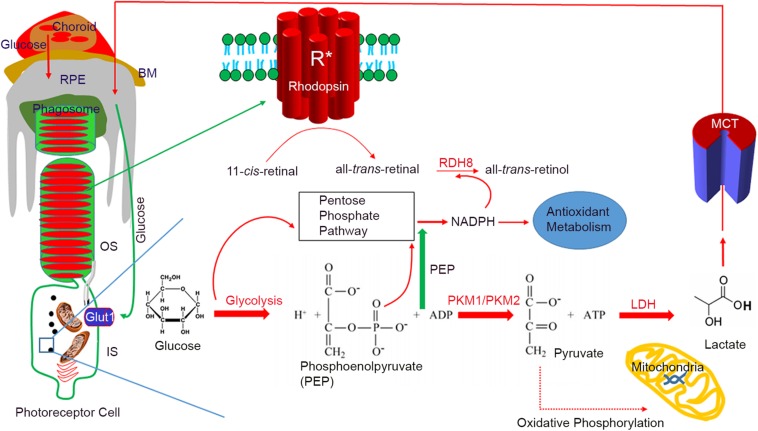
Aerobic glycolysis in photoreceptor functions. Photoreceptors are post-mitotic cells, interdigitated with retinal pigment epithelium (RPE). Glucose enters the RPE through choroidal circulation. Bruch’s membrane (BM) separates the RPE and choroid. Glucose from the RPE is transported to a photoreceptor through glucose transporter 1 (Glut1). In photoreceptor cells, the majority of glucose is redirected to anabolic processes. Every day by the onset of light, 10% of photoreceptor tips are phagocytosed by the RPE, and some of the digested lipids are recycled back to photoreceptor cells. A high rate of membrane synthesis takes place in photoreceptor cells. The redirected glucose is utilized for the anabolic processes, which include lipid synthesis, RNA/DNA synthesis, and protein synthesis. The NADPH generated through the pentose phosphate pathway (PPP) is used for lipid synthesis and reduction of all-*trans*-retinal to all-*trans*-retinol by the retinol dehydrogenase 8 (RDH8). NADPH is also needed for antioxidant metabolism. Photoreceptor cells express predominantly PKM2, while PKM1 is a minor protein. Pyruvate formed during glycolysis will be converted to lactate by lactate dehydrogenase (LDH). Lactate is transported to RPE through lactate transporters (monocarboxylate transporter), where it converts to pyruvate through LDH to fuel mitochondria. Glucose-mediated oxidative phosphorylation is minimal. PKM2 favors aerobic glycolysis and has a lower affinity for PEP, which results in the accumulation of PEP in the outer segments, which triggers the PPP. BM, Bruch’s membrane; RPE, retinal pigment epithelium; OS, outer segment; IS, inner segment; RDH8, retinal dehydrogenase 8; PEP, phosphoenolpyruvate; LDH, lactate dehydrogenase; PKM1, M1 isoform of pyruvate kinase; PKM2, M2 isoform of pyruvate kinase; Glut1, glucose transporter 1; MCT, monocarboxylate transporter.

In the photoreceptor cells, the dark current needs a large amount of ATP through the tricarboxylic acid (TCA) cycle. Photooxidation products generated through rhodopsin activation generate reactive oxygen species (ROS), which are toxic to the photoreceptor cell (Punzo et al., 2012; Adler et al., 2014). The pentose phosphate (PPP) pathway or hexose monophosphate (HMP) shunt, as the principal components of cellular anabolism, generate the NADPH used to reduce oxidized glutathione to reduced glutathione, which neutralizes the toxic effects of ROS (Figure 1). The PPP-generated NADPH is necessary for lipid biosynthesis (Punzo et al., 2012). During the photobleaching of rhodopsin, 11-*cis*-retinal is isomerized to all-*trans*-retinal; NADPH is an absolute requirement for the reduction of all-*trans*-retinal by the enzyme retinol dehydrogenase 8 (RDH8) (Figure 1).

Checkpoints in the glycolytic pathway and shunting the glucose to the PPP to generate NADPH and ribose are evolutionarily established (Jiang et al., 2014). Glucose redirection for anabolic processes is necessary for photoreceptors, as they require copious amounts of NADPH for disc membrane biogenesis, antioxidant metabolism, and reduction of all-*trans*-retinal as a means to detoxify this retinoid.

Although it was a century ago that Warburg noticed that tumor cells and retinal cells redirect glucose for anabolic processes, the mechanism behind the fuel redirection from oxidative phosphorylation to anabolic processes was not known until 2011. Investigators for the first time identified that higher pyruvate kinase enzyme activity results in the release of ROS during yeast respiration (Gruning et al., 2011). These investigators also observed that low enzyme activity of pyruvate kinase results in the accumulation of a substrate, phosphoenolpyruvate (PEP), that activates a negative feedback inhibitor of the PPP, triosephosphate isomerase (TPI1), which in turn activates the PPP. This activation results in the generation of NADPH for antioxidant metabolism and increased lipid synthesis (Gruning et al., 2011). These studies led other scientists to examine more closely the pyruvate kinases and their roles in energy metabolism. This study for the first time demonstrated that pyruvate kinase triggers a metabolic feedback loop that controls redox metabolism in respiring cells (Gruning et al., 2011).

Another landmark observation made by Dr. Lewis Cantley, that pyruvate kinase M2 isoform is a phosphotyrosine binding protein (Christofk et al., 2008b), suggested that pyruvate kinase could potentially undergo tyrosine phosphorylation. These observations, along with yeast studies, inspired several investigators to look more closely at the biological roles of pyruvate kinases.

### Pyruvate Kinase Isoforms

Pyruvate kinase is a known glycolytic enzyme involved in the last step of glycolysis by converting PEP to pyruvate. Four isoforms of pyruvate kinase have been identified: PKM1, PKM2, PKR, and PKL (Imamura and Tanaka, 1982). More than one isoform may be expressed in a single tissue, but individual cells may predominantly express one isoform (Imamura et al., 1972; Cardenas and Dyson, 1978; Israelsen and Vander Heiden, 2015). Tissues with high catabolic activity, such as muscle, heart, and the brain, predominantly express PKM1 (Israelsen and Vander Heiden, 2015). In the liver, PKL is the predominant isoform, while PKL expression is very low in the kidney (Israelsen and Vander Heiden, 2015). The expression of the PKR and PKL isoforms is restricted to certain tissues and cell types; PKR expression is restricted to red blood cells (Israelsen and Vander Heiden, 2015). There was a study conducted in 2016 shows the isoform expression of PKM1 and PKM2 in different mouse tissues. This study shows that heart, skeletal muscle, smooth muscle, brain predominantly express PKM1 whereas kidney, pancreatic islets, intestine, white fat, lung, lymphocytes, thymus, spleen, and ovaries predominantly express PKM2 (Dayton et al., 2016).

The PKM1 and PKM2 isoforms arise from a single gene as alternatively spliced products (Israelsen and Vander Heiden, 2015). The PKM gene is present in humans, mice, and rats, and each contains 12 exons (Noguchi et al., 1986; Takenaka et al., 1991). The lengths of exons 9 and 10 are identical; exon 9 is specific to PKM1, whereas exon 10 is specific to PKM2 (Clower et al., 2010). A properly spliced transcript has either exon 9 or exon 10 (Clower et al., 2010). To generate the PKM2 transcript, exon 9 must be repressed to include exon 10 in the final transcript (Clower et al., 2010). The repression of exon 9 is mediated by three splicing factors, the polypyrimidine tract binding protein (PTB), heterogeneous nuclear protein A1 (hnRNPA1), and heterogeneous ribonucleoprotein A2 (hnRNPA2), whereas binding of serine/arginine-rich splicing factor 3 (SRF3) to exon 10 promotes the inclusion of this exon in the transcript (Clower et al., 2010; David et al., 2010). The transcriptional regulation of PKM2 is well understood, but the transcriptional regulation of PKM1 is very limited. The lack of knowledge on PKM1 transcript production could be due to its expression to a lesser extent in differentiated tissues.

### Regulation of PKM1 and PKM2

**FIGURE 2:**
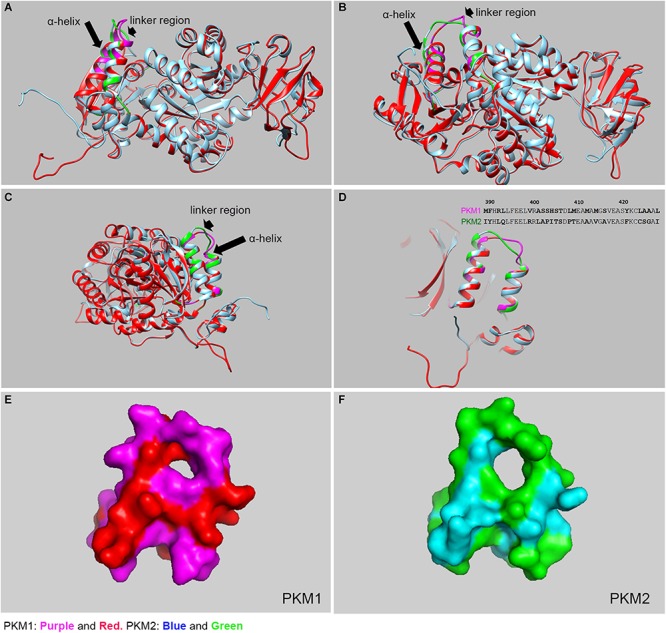
Structural comparison between mouse PKM1 and PKM2. The crystal structure of human PKM1 and PKM2 has been solved (PDB ID: PKM2, 4JPG, PKM1, 3SRF). We carried out the molecular modeling of mouse PKM1 and PKM2 using the Raptor X template-based protein structure modeling web server and aligned, with Clustal Omega, and the resulting structures were generated using the UCSF Chimera program and PyMOL (PyMOL Molecular Graphics System, Version 4.5 Schrödinger, LLC). The structural alignment of PKM1 and PKM2 at the different views **(A–C)**. The purple color represents PKM1 divergent region/residue and the red represents PKM1 conserved region/residue. The green color represents the PKM2 divergent region/residues and the blue represents PKM2 conserved region/residue. The majority of residues in PKM1 are identical to PKM2, except a region where diverge can be noticed in the linker region (see arrowhead) and 2 alpha helices (see arrow). A significant divergence was shown to be localized to residues 389–428 which consist of 2 alpha helices and joining linker region **(D)**. *inset:* Divergent protein sequence between PKM1 and PKM2 (389–428). The surface plots of PKM1 **(E)** and PKM2 **(F)**, respectively as seen in panel **(D)**.

PKM1 and PKM2 differ in only 22 amino acids in their sequences, but the functions mediated by PKM1 and PKM2 are distinct. Interestingly, PKM2 has >95% identity and >98% similarity in sequence with PKM1. The crystal structure of human PKM1 and PKM2 has been solved (PDB ID: PKM2, 4JPG, PKM1, 3SRF). We carried out the molecular modeling of mouse PKM1 and PKM2 using the Raptor X template-based protein structure modeling web server (de la Monte et al., 2003; Peng and Xu, 2011a, b; Ma et al., 2013) and aligned, with Clustal Omega (Sievers et al., 2011), the resulting structures using the UCSF Chimera program (Pettersen et al., 2004; Figure 2). The majority of residues in PKM1 are identical to PKM2. A significant divergence can be noticed in the two alpha helices and a linker region with 21 of the 22 of divergent residues being localized here (aa 389–428) (Figure 2). The other divergent residue is (Glu 433 PKM1; Lys 433 PKM2) is located away from this region and is situated on the c-terminal side of a β-sheet downstream of these two alpha-helices. Consistent with this divergence this region shown increased estimated root-mean-square deviation of atomic position (RMSD) values compared to the full-length protein and full-length protein lacking this divergent region. Upon removal of this linker region from the full-length protein exhibited a decreased RMSD value (Table 1) likely indicating that this region is different between these two isoforms at a structural level. The surface plot of this divergent region in PKM1 and PKM2 further shows distinct characteristic features between both isoforms. Further studies are needed to establish the importance of this region at a functional level.

**TABLE 1:** RMSD generated using Pymol standard algorithm.

**Region**	**Amino acids**	**RMSD (Å)**
Full-length protein	1–531	0.38
Divergent region (2α-helices)	389–428	0.54
Full-length protein excluding the divergent region	1–388, 429–531	0.33

*Values depicted in the table are after five rounds of outlier exclusion. Complete analysis is shown in Supplementary Material.*

These differences between PKM1 and PKM2 seem to be localized to a part of the protein which appears key for dimerization and/or tetramerization indicating these changes might affect the substrate specificity based on the extent oligomerization (Guo et al., 2013; Israelsen and Vander Heiden, 2015). PKM1 always exists as a stable constitutive tetramer, whereas PKM2 exists in between the tetrameric and dimeric configurations, depending on the extent of posttranslational modifications and allosteric activators (Iqbal et al., 2014; Prakasam et al., 2018; Wiese and Hitosugi, 2018). Fructose 1-6- bisphosphate (FBP) (Wang et al., 2017) is one of the glycolytic intermediates of glycolysis. It binds to PKM2 and promotes the tetrameric form and increases its affinity toward its substrate, PEP. This high enzyme activity catalyzes the conversion of PEP to pyruvate and promotes oxidative phosphorylation. PKM2 undergoes tyrosine phosphorylation on tyrosine-105 (Y105) by oncogenic tyrosine kinases, glucose-mediated acetylation on lysine-305 (K305), and oxidation of cysteine-358 (C358) residue by insulin-induced ROS (Hitosugi et al., 2009; Anastasiou et al., 2011; Lv et al., 2011). These posttranslational modifications favor PKM2 to be in the dimeric conformation, which makes the enzyme inactive. The inactive enzyme has a low affinity for PEP that results in the activation of the PPP to promote anabolic processes.

Previous studies showed that substitution of PKM2 in tumors with PKM1 reverses the cancer phenotype (Christofk et al., 2008a). These earlier studies showed that PKM1 favors oxidative phosphorylation, whereas PKM2 promotes anabolic processes (Dong et al., 2016).

The role of PKM1 in tumor progression is controversial. More recently, PKM1 has been shown to promote the growth of multiple tumor lines (Morita et al., 2018). These studies showed that PKM1 promotes the catabolism of glucose without altering the biosynthetic pathways. The tumor progression mediated by PKM1 occurs through PKM1-mediated activation of autophagy/mitophagy. For this tumor phenotype, PKM1, but not PKM2, supports malignant cell proliferation (Morita et al., 2018). Pyruvate kinase M2 activation has been shown to protect against the progression of diabetic glomerular pathology and mitochondrial dysfunction (Qi et al., 2017).

### Non-metabolic Functions of PKM2

PKM2 is known to regulate glycolytic activity and anabolic processes (Zhang et al., 2019). Besides, PKM2 has an intrinsic protein kinase activity and phosphorylates proteins on tyrosine, threonine, and serine residues (Gao et al., 2012; Israelsen and Vander Heiden, 2015). Interestingly, PKM2 uses ADP and PEP as phosphate donors, instead of ATP (Israelsen and Vander Heiden, 2015). PKM2 is also a transcriptional co-activator and mediates non-glycolytic nuclear function by phosphorylation (Luo and Semenza, 2012; Hsu and Hung, 2018). Oncogenic mediated tyrosine phosphorylation of PKM2 on Y105 results in the accumulation of glycolytic intermediates and redirects glucose for anabolic processes. Under physiological conditions, the dephosphorylation of PKM2-Y105 is mediated by protein tyrosine phosphatase 1B (Bettaieb et al., 2013; Prakasam et al., 2018). PKM2 undergoes phosphorylation on S37 by EGFR-activated extracellular signal-regulated kinase (ERK) 1/2. IGF-1R activated Akt phosphorylates PKM2 on S202, and proviral insertion in murine lymphoma 2 (PIM2) facilitates the nuclear translocation of PKM2 with the help of nuclear importin α5 (Prakasam et al., 2018). The nuclear-translocated PKM2 facilitates the transcriptional activation of β-catenin and signal transducer and activator of transcription 5 (STAT5), and enables the expression of several genes, including cyclin D1, lactate dehydrogenase (LDH), PKM2, glucose transporter 1 (GLUT1), and cMyc, to redirect the glucose metabolism that is essential for cancer progression (Prakasam et al., 2018). It was also shown that nuclear PKM2 regulates the Warburg effect (Yang and Lu, 2013).

### Pyruvate Kinase Isoforms in the Retina

The retina is a post-mitotic neuronal tissue consisting of seven layers of cells, including rod and cone photoreceptor cells. These cells are metabolic, and their energy expenditure is almost equivalent to that of a cancer cell. Glycolysis is indispensable for photoreceptor cell survival; ablation of this pathway resulted in retinal degeneration (Chinchore et al., 2017), whereas the upregulation of this pathway is neuroprotective (Zhang et al., 2016). Aerobic glycolysis is essential for normal rod function and prevents cone degeneration in retinitis pigmentosa (Petit et al., 2018). Shunting glucose to aerobic glycolysis regenerates cone outer segment synthesis (Wang et al., 2016, 2019). Furthermore, the rod-derived cone viability factor has been shown to promote cone photoreceptor survival by stimulating aerobic glycolysis (Ait-Ali et al., 2015). It has also been shown that stimulation of adenosine monophosphate-activated protein kinase (AMPK) by metformin protects photoreceptor and RPE in three mouse models of retinal degeneration (Xu et al., 2018). In cancer cells, metformin increases aerobic glycolysis and reduced glucose metabolism through the citric acid cycle (Andrzejewski et al., 2014) and might similarly protect the photoreceptors. Both rod and cone photoreceptor cells are close contacts with retinal pigment epithelial (RPE) and Müller cells. Choroidal circulation brings glucose to the retina. The glucose enters the RPE cell via glucose transporters and is then delivered to rod photoreceptor cells (Punzo et al., 2012; Hurley et al., 2015). Altered mitochondrial metabolism in the RPE is the prime cause of RPE dysfunction and age-related macular degeneration (AMD) (Morohoshi et al., 2012; Ferrington et al., 2017; Riazi-Esfahani et al., 2017; Fisher and Ferrington, 2018). RPE cells do not use glucose for metabolism. Through aerobic glycolysis, glucose is converted to lactate. Through lactate transporters (Adijanto and Philp, 2012), lactate is transported to RPE cells, where it can be converted to pyruvate by the action of LDH and then converted to pyruvate, fueling the mitochondria for oxidative phosphorylation (Figure 1). In this regard, the RPE cell is dependent on oxidative phosphorylation, whereas the photoreceptor cell is more dependent on glycolysis (Kanow et al., 2017). The lactate made in the photoreceptor cells is also transported to Müller cells, where it is converted to pyruvate, and fuels the mitochondria for oxidative phosphorylation. Thus, a metabolic ecosystem exists between RPE, photoreceptor, and Müller cells (Kanow et al., 2017).

The retina expresses both PKM1 and PKM2. However, the expression is cell-specific. PKM1 is predominantly expressed in the inner plexiform layer and ganglion cell layer, and weakly expressed in the rod inner segments (Rajala et al., 2016). PKM2 is predominantly expressed in the inner segments and outer plexiform layer of the photoreceptors and weakly expressed in the inner plexiform and ganglion cell layers (Lindsay et al., 2014; Rajala et al., 2016).

**FIGURE 3:**
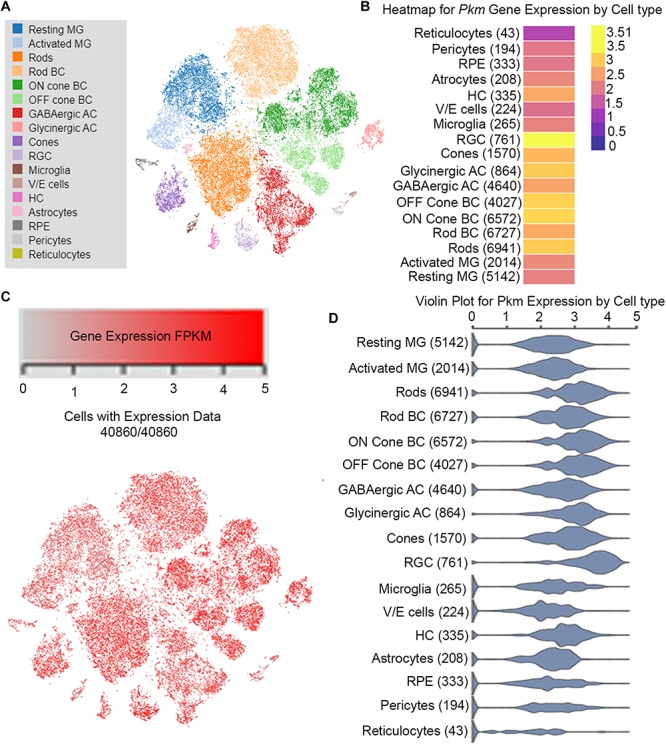
Cell-specific *Pkm* expression in adult mouse retina on single-cell RNA-seq analysis. From this publicly available database https://proteinpaint.stjude. org/F/2019.retina.scRNA.html, we searched the *Pkm* gene expression in various cell types of the retina from single-cell RNA-seq analysis **(A)**. t-distributed stochastic neighbor embedding (t-SNE) plots of gene expression distribution in adult mouse retina **(B)**. Each dot represents a single cell. Levels of gene expression of *Pkm* in different cell types **(C)**. FPKM: Fragments/Kilobase of transcript per Million mapped reads. Violin plots showing expression levels *Pkm* gene in different cell types of the mouse retina **(D)**.

To understand the gene networking that controls neuronal cell reprogramming in response to injury, a team of researchers has carried out single-cell RNASeq analysis from the chick, mice, and zebrafish (Hoang et al., 2019). Their transcriptomic and epigenetic analysis show that during injury several genes regulate the neuronal regeneration in the vertebrate retina (Hoang et al., 2019). These authors have deposited the data and created an interactive search at https://proteinpaint.stjude.org/F/2019.retina.scRNA.html. From this publicly available database, we searched the pyruvate kinase (*Pkm*) gene expression and found that it is expressed differently in various cell types of the retina (Figure 3). Consistent with our previous studies that pyruvate kinase expressed in both rod, cone and the inner retinal layer of the retina (Figure 3). This analysis does not differentiate the individual isoforms of either PKM1 or PKM2, but give in general the expression profile of *Pkm* gene. In photoreceptors, PKM2 undergoes tyrosine 105 phosphorylation in a light-dependent manner (Rajala et al., 2016), similar to cancer cells (Hitosugi et al., 2009). This phosphorylation has been shown to inhibit pyruvate kinase activity and supports the notion that reduced PKM2 activity promotes anabolic activity. Several investigators have deleted the PKM2 gene specifically in rod photoreceptor cells or performed pan-retinal deletion of PKM2 with short hairpin RNAs (shRNA) (Chinchore et al., 2017). A profound retinal degeneration phenotype was reported when PKM2 was knocked down with shRNA (Chinchore et al., 2017). Conditional deletion of PKM2 in rod photoreceptor cells resulted in a very slow retinal degeneration. However, by 5 months, knockout mice experienced reduced rod function (Rajala et al., 2018a). It is well known that exon 10 (PKM2) suppresses the expression of exon 9; this is consistent with the transcription repression reports that deletion of PKM2 in photoreceptor cells produces upregulation of PKM1 in these cells (Chinchore et al., 2017; Wubben et al., 2017; Rajala et al., 2018a). In rods, PKM1is a minor protein compared with PKM2 (Rajala et al., 2016). The structural and functional changes observed in PKM2-deleted rods could be due to the upregulation of PKM1. Interestingly, combined knockdown of PKM1 and PKM2 has been shown to shorten rod outer segments (Chinchore et al., 2017). This phenotype could be reversed by supplementing PKM2 cDNA, but not PKM1 cDNA (Chinchore et al., 2017). In the presence of endogenous PKM2, forceful expression of PKM1 has been shown to reduce the length of rod outer segments (Chinchore et al., 2017).

Since PKM1 knockout mice are not available, the functional role of PKM1 in the retina is currently unknown. Mouse rods lacking PKM2 showed upregulation of PKM1, yet failed to complement the reduced rod function (Rajala et al., 2018a). In the retina, PKM2 is present around 150 pmol and PKM1 is present around 26 pmol (Rajala et al., 2018a). In the absence of PKM2, there was a three-fold compensatory increase of PKM1 with 65 pmol of PKM1. PKM2 is a highly abundant protein in rods, and its concentration is very close to that of rhodopsin (Lyubarsky et al., 2004). It was previously reported that a single allele of rhodopsin gene gives 30 million rhodopsin molecules per rod; around 550–650 pmol rhodopsin per 6.4 million rods was estimated in wild-type mice with two alleles (Lyubarsky et al., 2004). In cancer cells, switching PKM2 with PKM1 reverses the cancer phenotype (Christofk et al., 2008a). One study found no evidence for a shift in PKM1 to PKM2 expression during tumorigenesis (Bluemlein et al., 2011).

Metabolic glucose flux experiments showed that loss of PKM2 resulted in the accumulation of glycolytic intermediates, with a decrease in pyruvate and lactate levels (Rajala et al., 2018a). In this regard, photoreceptors do not behave like cancer cells. The conversation of PEP to pyruvate is significantly slower in rod-specific PKM2 knockout mice. Even though the PKM1 is upregulated in rods lacking PKM2, there is less pyruvate kinase activity and accumulation of glycolytic intermediates. The accumulation of glycolytic intermediates in the absence of PKM2 suggests that PKM1 has a higher Km for PEP *in vivo* or that there is a lower level of pyruvate kinase activity. PKM1 upregulation in the outer retinas of PKM2 knockout mice showed increased expression of genes involved in glucose metabolism, which led to chronic degenerative changes in the outer retinas of PKM2-deleted mice (Wubben et al., 2017). These studies led to the hypothesis that reprogramming metabolism may be a novel therapeutic avenue for photoreceptor neuroprotection during stress conditions. Interestingly, the deletion of PKM2 in rods resulted in the accumulation of glycolytic intermediates, which also resulted in increased levels of NADPH (Rajala et al., 2018a). PKM2 is also expressed in cones. Mouse cones lacking PKM2 undergo early onset of cone degeneration (Rajala et al., 2018b).

### PKM2 Regulates the Photoreceptor-Specific Protein Expression

In mice, rods lacking PKM2 show decreased expression of cGMP-phosphodiesterase 6β (Pde6β) and GTPase-activating protein, regulators of G-protein signaling 9 (RGS9) (Rajala et al., 2018a). Increased levels of cGMP bind to the cGMP-gated channel and facilitate the influx of ions (dark current) that depolarize the photoreceptor (Kaupp and Seifert, 2002; Yau and Hardie, 2009). Pde6β hydrolyzes cGMP and regulates the cGMP-gated channel at the plasma membrane of the rod outer segments (Kaupp and Seifert, 2002). Decreasing the cGMP levels in the cell blocks the inflow of dark-current and the cell becomes hyperpolarized (Kaupp and Seifert, 2002). Changes in the levels of cGMP alter the phototransduction kinetics (Takemoto and Cunnick, 1990).

Pde6β is specific to photoreceptor cells. However, a 2013 study reported that aggressive breast tumors and breast cancer cell lines overexpress Pde6β (Dong et al., 2013). Interestingly, these tumors also expressed increased PKM2 (Israelsen et al., 2013). Consistent with the overexpression of PKM2 and Pde6β in tumors, photoreceptor cells lacking PKM2 express reduced levels of Pde6β (Rajala et al., 2018a). The reduced expression of Pde6β is due to a non-metabolic role of PKM2 as a transcriptional co-activator of Pde6β. Consistent with this notion, PKM2 increases Pde6β promoter activity *in vitro* (Rajala et al., 2018a). PKM2 can also indirectly regulate PD6β, as it requires Hsp90 and its co-chaperone AIPL for maturation (Aguila and Cheetham, 2016). It was reported previously that Hsp90 inhibition blocked Hsp90-AILP interaction results in PDE degradation (Xu et al., 2017). Further, mutations in the gene encoding AIPL1 cause Leber congenital amaurosis (LCA) (Dharmaraj et al., 2004). Hsp90 promotes cell glycolysis, proliferation, and inhibition of apoptosis by regulating PKM2 abundance via threonine-328 phosphorylation in hepatocellular carcinoma (Xu et al., 2017). Further, PKM2 directly interacts with Hsp90, suggesting that the PKM2 interaction with Hsp90 might regulate cellular levels of Pde6β. Further studies are needed to delineate the PKM2-Hsp90-Pde6β axis.

Mouse rods lacking PKM2 show accumulation of glycolytic intermediates and decreased levels of pyruvate and lactate, suggesting that less pyruvate kinase enzyme activity is available to generate pyruvate in the presence of increased expression of PKM1 (Rajala et al., 2018a). These studies raise an important open question in photoreceptor biology. That is, there is a question of how photoreceptors survive in the absence of glycolysis, as they are highly metabolic in the dark and require copious amounts of ATP. The possible sources of ATP to preserve photoreceptors until mice reach 5 months of age are also unknown.

The major source of pyruvate production is glycolysis. An alternative pathway to produce pyruvate has been reported (Vander Heiden et al., 2010). In this PEP pathway, the substrate of pyruvate kinase acts as a phosphate donor in the phosphorylation of the glycolytic enzyme phosphoglycerate mutase (PGAM1) in PKM2-expressing cells (Vander Heiden et al., 2010). PEP is transferred onto the catalytic histidine (His^11^) residue on human PGAM1. This reaction has been shown to occur with physiological concentrations of PEP and produced pyruvate in the absence of PKM2 activity. Pyruvate can also be produced through amino acid metabolism (Chang and Goldberg, 1978; Weber, 2001).

Upregulated PKM1 failed to complement PKM2 function in rods. Thus, there must be another source of acetate to fuel the TCA cycle to generate ATP. There is no potential source of ATP generation in PKM1-upregulated, PKM2-deleted rods. However, the source could be 3-carbon compounds, such as pyruvate and lactate, from Müller cells. Müller cells receive lactate from photoreceptor cells to fuel their mitochondria by converting the lactate to pyruvate through LDH (Kanow et al., 2017). PKM2 is not expressed in Müller cells under physiological conditions (Lindsay et al., 2014). However, when we analyzed a publicly available database of the single Müller cell transcriptome of the rhodopsin KO mouse showed that at the time points of rod- and cone-degeneration, PKM2 expression in the Müller cells is significantly higher than that in non-degenerating wild type mice (Roesch et al., 2012). Thus, it is evident that retinal degeneration induces the expression of PKM2 in Müller cells.

The PKM2 isoform is expressed in both rod and cone photoreceptors (Rajala et al., 2016), but cone photoreceptors lacking PKM2 undergoes rapid cone degeneration compared to rods lacking PKM2 (Rajala et al., 2018b). The rod degeneration is slow in mice lacking PKM2, however, they eventually degenerate, suggesting that other compensatory mechanisms might contribute to the survival of photoreceptor cells. The open question becomes how PKM2 knockout mouse retinas survive and the source of energy production in the retinas remains an open question. One possibility could be the utilization of amino acids for the conversion pyruvate. Consistent with this possibility, cysteine catabolism and serine biosynthesis pathways support the production of pyruvate during pyruvate kinase knockdown in pancreatic cancer cells (Yu et al., 2019). This possibility cannot be ruled out in mouse retinas lacking PKM2. In ischemia/reperfusion injury, pyruvate dehydrogenase kinase (PDK) inhibitor has recently been shown to inhibit retinal cell death and improves energy metabolism in rat retinas (Sato et al., 2020). PDK phosphorylates pyruvate dehydrogenase (PDH) and inhibits the conversion cytosolic pyruvate to mitochondrial acetyl-CoA, the substrate for the TCA cycle (Sutendra et al., 2013, 2014). PDK undergoes phosphorylation by tyrosine kinase receptor signaling and the phosphorylated form of PDK inhibits PDH, which results in the inhibition of mitochondrial oxidative phosphorylation (Sutendra et al., 2013, 2014). PDK inhibitors are commonly used to treat cancers to facilitate the conversion of pyruvate to acetyl CoA, which enters into the TCA cycle and produces ATP (Sutendra et al., 2013). A possibility cannot be ruled out that PKM2 deletion may inhibit the phosphorylation of PDK that might promote the oxidative phosphorylation, which may keep the retinas stay alive for some time. If this idea holds, modulating PKM2 levels may promote photoreceptor survival in retinas that are predetermined to degenerate. Especially, in the degenerating retinas (for example, mouse models of *retinitis pigmentosa*), reducing the anabolic activity and redirect the pyruvate to fuel mitochondria for oxidative phosphorylation may prevent retinal cell death and promote retinal cell survival.

Another possibility is the β-oxidation of fatty acid-generated acetate in the mitochondria. A 2016 study reported that photoreceptors utilize both fatty acids and glucose for ATP production, and defects in the fatty acid transport pathway were shown to cause AMD (Joyal et al., 2016). If this pathway is active, then there would be an inflow of fatty acids from other cells at a higher level for membrane and outer segment renewal processes. We know from the literature that upon phagocytosis of rod outer segments in the RPE, the n3 and n6 polyunsaturated fatty acids (PUFA) are recycled back to photoreceptor inner segments for rod outer segment membrane phospholipid synthesis (Bazan et al., 1986; Stinson et al., 1991). These studies suggest that there could be a mechanism by which large quantities of fatty acids are delivered to inner segments from the RPE for oxidative production of ATP and membrane synthesis.

Based on this published work, we can hypothesize that some of the fatty acids recycled to inner segments from the RPE come from the choroidal circulation, and the inner segments may receive more fatty acids than are needed for membrane synthesis. The remaining fatty acids could be oxidized in the mitochondria to generate acetate to fuel the TCA cycle. Nevertheless, it has been shown that the n3 PUFAs is an indispensable constituent of outer segment membranes (Benolken et al., 1973; Wheeler et al., 1975). If these n3 PUFAs are utilized for acetate generation, their levels in the outer segments would be significantly decreased in diets that are deficient in PUFA precursors. This does not happen *in vivo* (Anderson and Maude, 1972), suggesting that there might be two pools of fatty acids in the inner segments: the PUFAs needed for membrane synthesis and another pool that could be used in the mitochondria for β-oxidation, especially shorter chain saturated and mono-unsaturated fatty acids that are directed to the mitochondria for β-oxidation.

We must keep in mind that we are dealing with a chronic, steady-state adaptive response to ablation of a gene, rather than an acute response to events that may have occurred over a matter of days. Thus, we are measuring responses caused by the long-term loss of the normal source of pyruvate for the TCA cycle’s production of ATP. In the retina, NADPH serves three important functions: reducing all-*trans*-retinal to all-*trans*-retinol, reducing oxidized glutathione, and providing reducing equivalents for lipid and protein synthesis. The key to understanding in PKM2 knockout mice retinas and how these mice survived and the source of energy production in these retinas remain open questions.

## Conclusion

Studies in the retina show that aerobic glycolysis is essential for retinal cell survival. Although photoreceptors are post-mitotic, PKM2 is the predominant isoform in these cells. In tumor cells, the substitution of PKM2 with PKM1 reversed the tumor phenotype. In photoreceptor cells, the deletion of PKM2 upregulates PKM1, yet it fails to clear the accumulation of glycolytic intermediates. Studies of retinal photoreceptor cells show that PKM2 is required for rod and cone photoreceptor function and survival and that PKM2 regulates photoreceptor cells both metabolically and non-metabolically.

## Author Contributions

The author confirms being the sole contributor of this work and has approved it for publication.

## Conflict of Interest

The authors declare that the research was conducted in the absence of any commercial or financial relationships that could be construed as a potential conflict of interest.
