# Supplementary material for: Aerobic Glycolysis in the Retina: Functional Roles of Pyruvate Kinase Isoforms
Source: Front Cell Dev Biol. 2020 Apr 30;8:266. doi: 10.3389/fcell.2020.00266 (PMC7203425; doi:10.3389/fcell.2020.00266)
Supplement: Supplementary file 1 [file Data_Sheet_1.PDF]

Full-length protein

```
Match: read scoring matrix.
 Match: assigning 531 x 531 pairwise scores.
 MatchAlign: aligning residues (531 vs 531)...
 MatchAlign: score 2596.000
 ExecutiveAlign: 531 atoms aligned.
 ExecutiveRMS: 16 atoms rejected during cycle 1 (RMSD=3.25).
 ExecutiveRMS: 18 atoms rejected during cycle 2 (RMSD=0.95).
 ExecutiveRMS: 35 atoms rejected during cycle 3 (RMSD=0.70).
 ExecutiveRMS: 27 atoms rejected during cycle 4 (RMSD=0.56).
 ExecutiveRMS: 25 atoms rejected during cycle 5 (RMSD=0.46).
 Executive: RMSD =    0.383 (410 to 410 atoms)
 Executive: object "aln_533698_to_533699" created.
```

Divergent region (2 a-helices)

```
 Match: read scoring matrix.
 Match: assigning 40 x 40 pairwise scores.
 MatchAlign: aligning residues (40 vs 40)...
 MatchAlign: score 94.000
 ExecutiveAlign: 40 atoms aligned.
 ExecutiveRMS: 3 atoms rejected during cycle 1 (RMSD=1.22).
 ExecutiveRMS: 2 atoms rejected during cycle 2 (RMSD=0.95).
 ExecutiveRMS: 2 atoms rejected during cycle 3 (RMSD=0.77).
 ExecutiveRMS: 2 atoms rejected during cycle 4 (RMSD=0.67).
 ExecutiveRMS: 1 atoms rejected during cycle 5 (RMSD=0.57).
 Executive: RMSD =    0.536 (30 to 30 atoms)
```

Full-length protein excluding the divergent region

```
Match: read scoring matrix.
 Match: assigning 477 x 477 pairwise scores.
 MatchAlign: aligning residues (477 vs 477)...
 MatchAlign: score 2427.000
 ExecutiveAlign: 477 atoms aligned.
 ExecutiveRMS: 8 atoms rejected during cycle 1 (RMSD=1.15).
 ExecutiveRMS: 30 atoms rejected during cycle 2 (RMSD=0.72).
 ExecutiveRMS: 28 atoms rejected during cycle 3 (RMSD=0.58).
 ExecutiveRMS: 23 atoms rejected during cycle 4 (RMSD=0.46).
 ExecutiveRMS: 15 atoms rejected during cycle 5 (RMSD=0.38).
 Executive: RMSD =    0.333 (373 to 373 atoms)
 Executive: object "aln_R1_to_R2" created.
```
